# Using the Laney p' Control Chart for Monitoring COVID-19 Cases in Jordan

**DOI:** 10.1155/2022/6711592

**Published:** 2022-09-19

**Authors:** Mazen Arafah

**Affiliations:** The Department of Industrial Engineering, The University of Jordan, Amman, Jordan

## Abstract

In this research, we examine the use of the Laney p' control chart and the application of test rules to assess governmental interventions throughout the COVID-19 pandemic and understand how certain activities and events that took place affected the infection rate. Data for the infection rate (IR) were collected between October 31, 2020, and March 19, 2022. The IR was calculated by dividing the number of confirmed cases by the number of PCR (polymerase chain reaction) tests performed. The IR data were subsequently plotted on the Laney p' control charts using the Minitab software. The charts thereby allowed us to study the effects on infection rates of the government's moves to restrict the movements and activities of the population, as well as the results of easing these restrictions. The restrictive measures proved to be effective in decreasing the infection rate, whereas relaxing these measures had the opposite effect. Typically, test signals are considered as an indication of a change in a process, although in some situations we have observed that slight changes are not accompanied by a signal. Regardless, the analysis shows cases where using test rules rapidly detected patterns and changes in IR, and allowing remedial action to be taken without delay. In this study, we use the Laney p' control chart to monitor the COVID-19 IR and compare its performance with that of the EWMA control chart. In addition, we analyze the performance of various test rules in detecting IR changes. Comparing the Laney p' control chart with the EWMA control chart, the data showed that in most cases, the Laney p' control chart was able to identify the change of IR faster. Comparing the performance of different tests in detecting changes in the IR, one can see that no particular test outperformed the others in all cases. We also recommend analyzing the data points in both single-stage and multistage analyses in accordance with this new perspective rather than the traditional one used in process improvement projects. Accordingly, the single-stage analysis gives a complete picture of how the infection rate is changing overall, whereas the multistage analysis is more sensitive to small changes.

## 1. Introduction

On March 11, 2020, the World Health Organization (WHO) declared the COVID-19 virus infection a pandemic [1]. As a result, countries worldwide dealt with the crisis under various uncertainty scenarios amidst the rise of new variants and tried to predict what would happen in 2021 [2–4], while the situation posed new challenges for policymakers everywhere.

Now, after two years of dealing with the pandemic, we can look at the different approaches adopted to tackle COVID-19 to learn what works best in controlling the virus.

**Figure 1:**
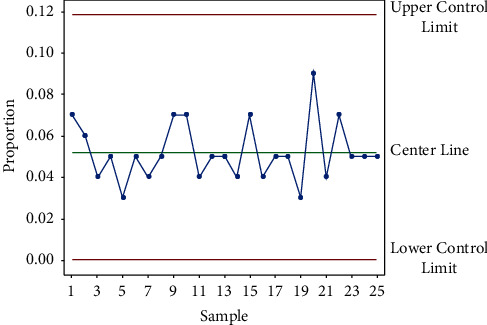
Typical control chart.

Control charts play a crucial role in better understanding processes and in monitoring and analyzing historical data. Devised by Dr. Walter A. Shewhart of Bell Telephone Labs in the 1920s, control charts were first utilized to address the variation problem in the manufacturing process to improve efficiency and reduce costs [5, 6]. Control charts are basically run charts—data plotted over a certain period of time [7]—with a mean line and control limits (see an example in Figure 1). The key quality characteristic is represented on the *y* (vertical)-axis, and time is represented on the *x* (horizontal)-axis. The control limits are the boundaries shown on the chart within which the given process can operate [8, 9]. The upper and lower control limits are generally set at three sigmas, and the mean is set at the midpoint of the control limits [10, 11]. The control limits are computed statistically, based on probability distributions such as the Gaussian, Poisson, or binomial distribution [12] (see Figure 1).

Control charts have long been used to distinguish between common cause variation and special cause variation [13]. The first type, which belongs to the category of chance or random variations, is the sum of the multitude of effects of a complex interaction of random or common causes, many of which are only slight. When special causes of variation are present, the variation will be excessive, and the process is classified as unstable, out of statistical control, or beyond the expected random variations [14]. Certain control charts can also assess both types of variations. These variations reflect the variations intrinsic in the process due to the many details that cannot be controlled precisely.

Control limits are warning signs that tell us variously [15]:

- Carry on or do nothing (stable zone—common causes of variation only).
- Be careful and seek more information since the process may be showing special causes of variation (warning zone).
- Take action, investigate, or, where appropriate, adjust the process (action zone—special causes of variation present).

There are several types of control charts, each of which can be used to chart a number of different characteristics. Among these, *variables' charts* are used when the characteristics can be objectively and quantitatively measured, whereas *attribute charts* are used when the characteristics can be counted [16]. Variables' control charts may be kept for individuals, averages, ranges, and standard deviations. Control charts for attributes show how characteristics normally vary over time, such as the percentage of defective attributes or defects per unit [17].

Although control charts were first used in industry, other service sectors, including health care, soon adopted them, with a number of applications of statistical process control (SPC) charts subsequently reported in medical settings (for the early papers citing the use of SPC in health care, see [18–23]; some of the recent ones are shown in [24, 25]).

In their literature review on the use of control charts in health care, Suman and Prajapati [26], for example, identified 40 research papers of interest from the 142 they initially examined. The authors classified the papers into the categories of emergency, surgery, epidemiology, radiology, cardiology, pulmonary, administration, and pharmaceutical. Each study detailed the types of study, the types of charts and variables used, and the country involved.

Woodall [27] furthermore discussed how the use of control charts in health-related applications differed from their use in industrial practice. Attribute data, for instance, are much more prevalent in healthcare applications than in industrial practice. Attribute data are binary in nature in that the initial response of an inspection activity yields only two possible outcomes: the unit of inspection either conforms to a requirement or it does not—a simple yes or no. The response is discrete. The *p*-chart is based on the binomial distribution [28].

More complex charts that accumulate information over time include the exponentially weighted moving average (EWMA) chart [29]. There have been a number of applications of the EWMA chart reported in the medical literature [24]. Time-weighted charts have the ability to detect small changes in the measure on the chart [30, 31].

Hospital epidemiologists are often concerned with outbreaks or other acute deviations from the norm, which, in continuous quality improvement (CQI) parlance, are referred to as the “special cause variations” already mentioned [20].

Benneyan [32] summarized several similarities between the concepts and terminology of epidemiology and SPC. Hospital epidemiology programs, for instance, tend to be concerned with both epidemic (nonsystemic) and endemic (systemic) infections, which in SPC terminology equate to unnatural (special cause) and natural (common cause) variability, respectively. Surveillance programs focus on the detection of sentinel and epidemic events (i.e., monitoring for unnatural process variation). However, the more pressing epidemiological concern of reducing endemic occurrences equates to the quality control efforts to improve a process whose defect rate, though in a state of statistical control, is still unacceptably high. The author also discussed how SPC charts could be used to identify special cause (atypical, nonendemic) events [33].

In brief, the research studies included both clinical- and medical-related performance measures. Among them, the following were included:

- the presence or absence of specific adverse postoperative outcomes [34];
- the rate of surgical site infections and 30-day mortality rates [35];
- the infection rates of ventilator-associated pneumonia (VAP) [36];
- the number of hospital-associated infections [37, 38]; and
- inpatient death, intensive care stay, reoperation, and severe complications within 30 days after surgery [39].

The nonclinical process performance measures included the following:

- the number of patients leaving the emergency department [22];
- admission time and length of stay [10];
- patient waiting time for obstetrics and gynecology clinic appointments [40];
- patient discharge time [41]; and
- patient waiting time in a pharmacy [42].

The bulk of studies using control charts in an epidemiological context have focused on infection control and hospital epidemiology, but there is a relatively sparse literature based on the use of control charts in public health data [43]. Control charts are still not part of standard public health practice [44, 45].

According to [24], control charts have been recently utilized to understand the unusual patterns in public health data. More specifically with regard to COVID-19, Perla et al. [43] stated: “Shewhart charts should become a standard method for learning from data in the context of a pandemic or epidemic.”

Parry et al. [46] designed a hybrid Shewhart chart to model three phases of an epidemic: pregrowth, growth, and postgrowth. They used chart to describe four “epochs”: pre-exponential growth, exponential growth, plateau or descent, and stability after descent, of the COVID-19 epidemic that emerged by incorporating a c-chart and I-chart with a log-regression slope. Perla et al. [43] used the same concept (hybrid c-chart and I-chart) to detect within a geographic area at the start and end of exponential growth in reported deaths. They used case studies and simulation to evaluate chart performance. The hybrid chart detected the start of exponential growth and identified early signals that the growth phase was ending.

Velayati et al. [47] used a funnel chart, an adaptation of the Shewhart p-chart, to compare between case fatality rates of COVID-19 for all 67 counties in Alabama State, USA. They concluded that funnel charts reliably identify counties with unexpected high and low COVID-19 case fatality rates.

Mahmood et al. [48] employed the *c* and exponentially weighted moving average (EWMA) control charts to monitor the number of reported deaths in Pakistan due to the COVID-19 pandemic. They were able to identify the pregrowth, growth, and postgrowth phases.

Without a scientific and reliable method to understand whether the variation in outcomes is attributed to meaningful signals of change (corrective actions or certain events) rather than variability we would expect, policymakers, care providers, and the public will be struggled to recognize whether conditions are improving. During a pandemic, timely and reliable signals that the number of reported infection cases is rising or falling may have enormous implications on public health and economics. Control charts given their ease of use and interpretability in real time offer a means to represent and interpret variation that will be essential for a successful response to pandemics.

In this study, we use the Laney p' control chart to monitor the COVID-19 infection rates and compare its performance with that of the EWMA control chart. We also examine the application of the SPC test rules and investigate the effect of various government precautionary decisions and actions, as well as various events, such as lockdowns, on the infection rate. We subsequently evaluate which tests are the most effective in detecting changes quickly.

The paper is organized as follows: Section 2 describes the methodology followed in this research; Section 3 presents the discussion of the results; and finally, Section 4 presents the conclusions.

## 2. Materials and Methods

**Figure 2:**
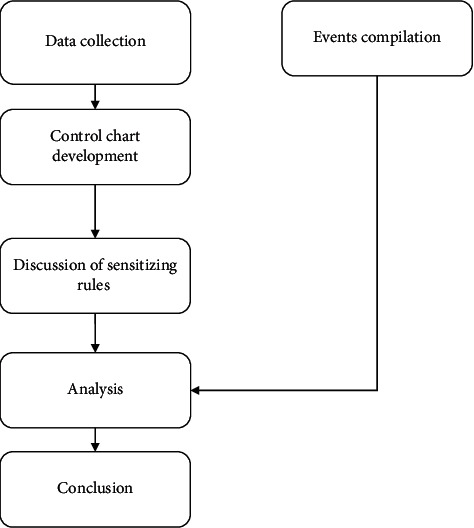
. Research methodology.

Figure 2 presents the steps followed in conducting this research. The following subsections describe each step in detail (Figure 2).

### 2.1. Data Collection

All the data used for this study correspond to those available both in public and in open sources. Data (the number of confirmed cases and a number of tests performed) were initially obtained from Appel et al.. The dataset includes several statistics, the number of confirmed cases and the number of tests performed. The data were recorded daily. Since some data were missing (number of confirmed cases or number of tests performed) for certain dates, the authors referred to press releases [49] to fill in the gaps and also to confirm the accuracy of the data obtained from [50] through random checks for some days. Prior to October 31, 2020, the Jordan Ministry of Health (MOH) recorded only the number of confirmed cases. The ministry also reported the number of tests performed from that date onward. The MOH monitors the rates per week (pandemic week), with each week starting on Saturday. The weeks are numbered, and Saturday, October 31, 2020, for example, is identified as PW 44–20; Saturday, January 2, 2021, is labeled as PW 1–21. The authors collected the data up to March 19, 2022.

### 2.2. Control Chart Development

As the focus of this research is on the percentage of confirmed cases from the PCR tests performed, the most appropriate control chart is the *p*-chart.

#### 2.2.1. p-Chart

The p-chart monitors the proportion of nonconforming items, that is, the fraction nonconforming or fraction defective, which indicates the infection rate (IR). In this case, it is a fraction of the cases confirmed by PCR tests.

The centerline ($\bar{p}$—the average fraction defective) is obtained by the following formula:

(1) $$\begin{array}{c} CL=\bar{p}=\frac{\overset{m}{\underset{i=1}{\sum }}D_{i}}{\overset{m}{\underset{i=1}{\sum }}n_{i}}\text{,} \end{array}$$

where *D*_*i*_ represents the nonconforming units in sample *i*, *n*_*i*_ is the number of items per sample *i* (sample size), and *m* is the number of samples.

The control chart limits are obtained by the following formula:

(2) $$\begin{array}{c} UCL/LCL=\bar{p}\pm 3\sqrt{\frac{\bar{p}\left(1-\bar{p}\right)}{n_{i}}}\text{.} \end{array}$$

#### 2.2.2. The Laney p' Control Chart

One needs to be careful when using the *p* control chart, especially when sample sizes are very large, as overdispersion may occur. In fact, Laney [51] cautioned about overdispersion, as this can cause the control limits to be too close together, leading to the identification of an “inappropriately” large number of data points signaling special cause variation. To prevent this occurrence, he proposed the Laney p' control chart [51].

The upper and lower control limits (UCL and LCL) are given by

(3) $$\begin{array}{c} \frac{UCL}{\;LCL}=\bar{p}\pm 3\sigma_{pi}\sigma_{z}\text{,} \end{array}$$

where

(4) $$\begin{array}{c} \sigma_{pi}=\sqrt{\frac{\bar{p}\left(1-\bar{p}\right)}{n_{i}}}\text{,}\\ \sigma_{z}=\frac{\bar{R^{\prime }}}{1.128}\text{,}\\ \begin{array}{c} {\bar{R}}^{\prime }=\frac{1}{k-1}\overset{k}{\underset{i=2}{\sum }}R_{i}^{\prime },\\ R_{i}^{\prime }=\left|z_{i}-z_{i-1}\right|\left(i=2,\ldots ..,k\right),\\ z_{i}=\frac{p_{i}-\bar{p}}{\sigma_{pi}}. \end{array} \end{array}$$

The Laney p' control chart is a widely used healthcare quality monitoring that has a very large sample size [31, 52–54]. Ahsan et al. [55] compared the performance of the Laney p' control chart with that of the conventional *p* control chart based on graphic visualization and average run length (ARL) criteria. They concluded that the *p*-chart becomes extremely oversensitive and cannot be used to monitor the process, while the p' control chart gives realistic results.

Typically, the control limits are initially calculated based on a historical set of data, as these limits are then used for ongoing monitoring as new data are collected and plotted. The retrospective analysis of historical data is referred to as Phase I, whereas the prospective monitoring of future data is referred to as Phase II [56].

The centerline and the upper and lower control limits are recalculated with each successive data point that is added to the control chart. We created the control charts using the Minitab software [57].

#### 2.2.3. Exponentially Weighted Moving Average (EWMA)

The EWMA chart statistic is a weighted moving average of current and past individual outcomes and is updated with each procedure. The weight is exponential, meaning that the contribution of past observations decreases going back in time [29].

The EWMA statistic (*z*_i_) is defined as follows:

(5) $$\begin{array}{c} z_{i}=\lambda p_{i}+\left(1-\lambda \right)z_{i-1}\left(i=1,\ldots ,m\right)\text{,} \end{array}$$

where *p*_*i*_ is the data point, and *λ* is a constant selected for the chart. The EWMA chart depends on the selected value of *λ*, where a smaller value of *λ* leads to quicker detection of small shifts [58]. Experience with EWMA charts suggests that a *λ* value between 0.1 and 0.3 gives the best performance [24]. *z*_*0*_ is usually set as the average of the series. The centerline ($\bar{p}$) is obtained by formula (1). The upper and lower control limits (UCL and LCL) are given by the following formula:

(6) $$\begin{array}{c} \frac{UCL}{LCL}=\bar{p}\pm 3\sqrt{\frac{\bar{p}\left(1-\bar{p}\right)\lambda \left[1-{\left(1-\lambda \right)}^{2i}\right]}{2\lambda }}\text{.} \end{array}$$

### 2.3. Sensitizing Rules

As stated above, one of the main objectives of utilizing control charts is to determine, based on the movement of the points, when a process is out of control so that necessary actions can be taken. A control chart may be said to display a lack of control under a variety of circumstances, any of which can provide some evidence of nonrandom behavior [59] and can indicate changes in the process average or spread.

Initially, Shewhart [60] defined a process as “out of control” when a single point was plotted outside the control limits. The Western Electric Company [61] proposed criteria to evaluate a process and detect the presence of unusual patterns such as runs or trends. Later on, several other criteria were added [62], which are known either as *supplementary rules* [33] or as *sensitizing rules* [63], as seen in Table 1. A control chart may be said to display a lack of control under a variety of circumstances, any of which can provide some evidence of nonrandom behavior and can indicate changes in the process average or spread. The main objective of this set of rules was to increase the sensitivity of the Shewhart charts and improve their potential to detect nonrandom patterns [64]. The rules are summarized in Table 1. For a good discussion of some of these rules, see [65].

Although some industries use all of these rules, only a subset of the rules are typically used in health care [7]. According to research [66], rules 2, 3, and 4 are widely recommended. Minitab 20 [57]performed rules 1, 3, 5, and 7 (shown in Table 1) on attribute data. These rules are reproduced in Table 2 using the Minitab numbering scheme.

Rules 2–4 are consistent in the sense that the chance of occurrence of each rule in a stable (in-control) process is approximately equal to the chance of Rule 1 occurring in a stable process [14]. There follows a brief discussion of each test rule.

#### 2.3.1. Rule Test 1

A single point outside the control limits will trigger a signal from this test. As mentioned earlier, the control limits are usually set at three standard deviations above and below the mean. The test signals an increase or decrease in the rate. The disadvantage of this test, however, is its lack of sensitivity to small shifts in the mean [67, 68].

#### 2.3.2. Rule Test 2

This test detects a run of eight consecutive points on one side of the centerline, where a run is defined as one or more consecutive data points on the same side of the centerline [69]. The number of consecutive data points varies from reference to reference [28].

Researchers [70, 71] have suggested variants of this rule, including the following:

- Whenever 11 successive points occur on the control chart, at least 10 will be on the same side of the centerline.
- Whenever there are 14 successive points on the control chart, at least 12 will be on the same side of the centerline.
- Where 17 successive points occur on the control chart, at least 14 will be on the same side of the centerline.
- In the case of 20 successive points on the control chart, at least 16 will be on the same side of the centerline.

#### 2.3.3. Rule Test 3

**Figure 3:**
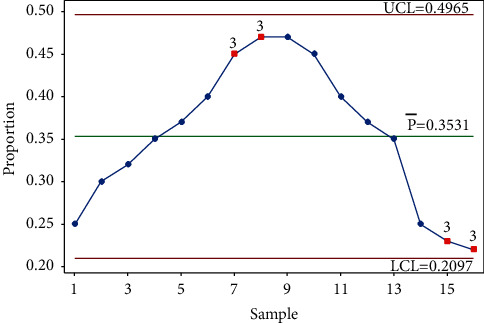
Test 3.

This test, which indicates 6 points in a row—all increasing or decreasing—is usually known as the *trend rule* [68]. As such, the test covers two cases. A gradual movement of points toward a *control limit is called a trend.* Trends moving up or down indicate that the process is changing [72] (see Figure 3).

The case of points in a row, either all increasing or decreasing, is also known as a run-up or run-down [73]. Six decreasing points in a row indicate an improvement and show that the rate of infection or the spread of the virus is under control, signaling that the actions taken are paying off. However, 6 points all increasing can act as an alarm, showing that the virus is spreading and the number of cases is rising, signaling that action needs to be taken.

Two issues need to be raised regarding the application of this rule. First, there is some inconsistency in the literature as to how many successive points should all increase or all decrease in value before a signal is produced by the chart [68]. The choice of six points is the most common to define a trend [65, 74], although some authors suggest using seven [11, 14, 75]. This test is the same rule used with a run chart, for which Provost and Murray [24] recommended using five points. Oakland and Oakland [14] referred to this as a warning signal. Note that using five points increases the chance of false signals, known as committing a type 1 error, i.e., signaling that a process is out of control when, in fact, it is not.

Minitab 20 [57] gives the user the option to choose the number of points.

**Figure 4:**
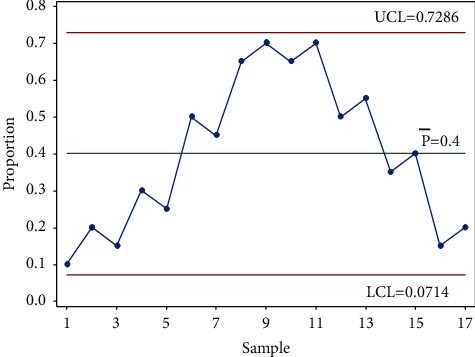
Sloping sawtooth pattern.

Second, if the drift in the rate is small, then the probability of six or seven consecutive points all increasing or decreasing is very small. Hence, AIAG [75] explained that for seven points in a row to consistently increase or decrease, they can either be equal to, greater, or lesser than the preceding points. Even so, it is possible to produce a sloping sawtooth pattern of points like the one shown in Figure 4.

For this case, the Western Electric Company [61] stated that a trend could be present if a high proportion of successive points were increasing/decreasing, but the company failed to define “a high proportion.” In this case, most software [57] will not trigger a signal but will result in a missed signal. A signal has to be detected visually. It would be helpful to suggest some criteria similar to those suggested for the run rule presented earlier.

#### 2.3.4. Rule Test 4

This test to indicate 14 points in a row, alternating up and down, was not identified.

However, Stapenhurst [76] emphasized that the rules given above are simply guidelines, indicators of a special cause of variation. He added that these guidelines should be used as more of a confirmation than as a rule to be rigidly followed.

An important remark is that in the EWMA chart, the plotted statistics are not independent from subgroup to subgroup, and thus, only Rule 1 can be used to detect special causes.

## 3. Results and Discussion

In order to provide the decision makers with real-time feedback, and as quickly as possible without any delays, the IR of each pandemic week was added to the control chart as at the control soon as it became available. We were not looking chart as a completed picture; instead, we observed it as the data evolved with each addition of information.

### 3.1. Charts' Development

**Figure 5:**
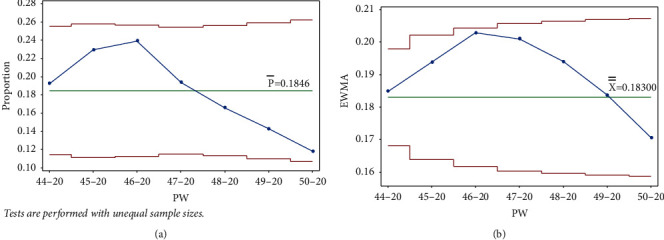
PW 50–20. (a) Laney p' control chart and (b) EWMA chart.

As mentioned earlier, data collection started in PW 44–20. The pandemic reached its first peak (in PW 46–20), following the general parliamentary election the previous week, on Tuesday, November 10, 2020 (PW 45–20). In response, the government enforced a general curfew starting at 10 : 00 pm on election night and ending on the morning of Sunday, November 15, 2020, at 6 : 00 am. This measure to contain and prevent the virus's possible spread due to gathering crowds affected a downward trend between PW 47–20 and PW 4–21. It follows that no test signal was triggered when drawing the control charts (Laney p' and EWMA) up to PW 50–20, as seen in Figure 5.

**Figure 6:**
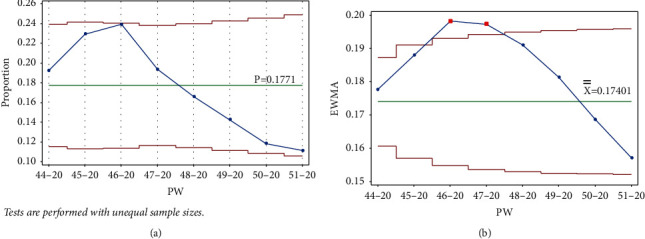
PW 51–20. (a) Laney p' control chart and (b) EWMA chart.

Once PW 51–20 was added, test 1 was triggered on PW 46–20 and PW 47–20 for EWMA chart only (Figure 6).

**Figure 7:**
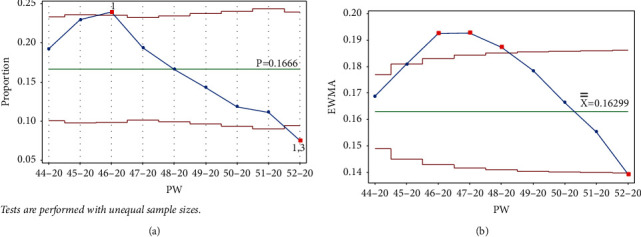
PW 52–20. (a) Laney p' control chart and (b) EWMA chart.

Once PW 52–20 was added, tests 1 and 3 were triggered in the same week, and test 1 was triggered on PW 46–20 for the Laney p' control chart. For the EWMA chart, test 1 was triggered in the same week and on PW 48–20.  Figure 7 clearly indicates the decreasing rate of infection, as the UCL and LCL are functions of the centerline (the mean); thus, adding more points below the centerline reduces the mean.

**Figure 8:**
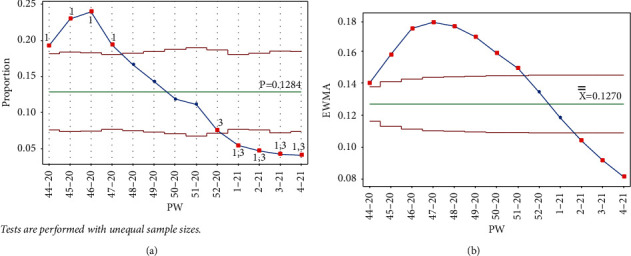
PW 4–21. (a) Laney p' control chart and (b) EWMA chart.

The addition of each of the data points PW 1–21, PW 2–21, PW 3–21, and PW 4–21 triggered tests 1 and 3 for the week, and test 1 in PW 45–20, PW 47–20, and PW 44–20, respectively, for the Laney p' control chart. As seen in Figure 8(a), test 1 signal was no longer present for PW 52–20, and the point was no longer out of control, indicating that as the mean was decreased, the IR was decreasing. More points from the UCL side were labeled as out of control, showing a slow decrease in the IR, which reached the lowest level in PW 4–21. Similar behavior is seen in Figure 8(b) for the EWMA chart.

**Figure 9:**
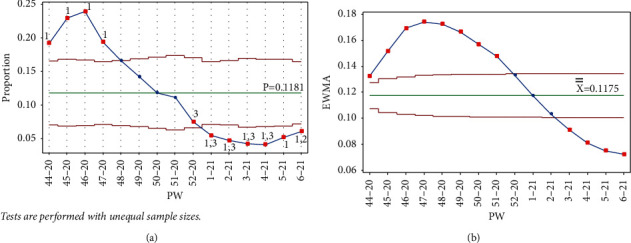
PW 6–21. (a) Laney p' control chart and (b) EWMA chart.

Adding point PW 5–21 triggered test 2, but did not trigger test 3, thus indicating a shift in direction as the IR began to increase, although it was not a big jump. Nonetheless, this should be seen as a signal that the situation was changing. Despite this short jump, the absence of test 3 and the presence of test 2 together showed a slow decrease in the IR overall. It is worth mentioning that in PW 52–20, cases of the Alpha variant were discovered in Jordan. In addition, starting PW 2–21, the government relaxed some of the restriction measures, lifting the Friday curfew and reducing the weekday curfew by two hours. The IR was still decreasing but at a slower pace, and it took about 4 weeks for the IR to change direction and start to increase (Figure 9(a)). Note that the EWMA chart did not signal this jump in the IR (Figure 9(b)).

When, in PW 5–21, certain recreational facilities were allowed to reopen and then in PW 6–21, a hybrid mode of teaching was subsequently introduced for grades from KG to Grade 3, and for Grade 12. The relaxation of restrictions had a dramatic effect on the IR. These actions, alongside the appearance of the Alpha variant, escalated the IR in the period from PW 5–21 to PW 11–21. PW 7–21 triggered test 2, but test 1 was not triggered, indicating a rise in the IR.

**Figure 10:**
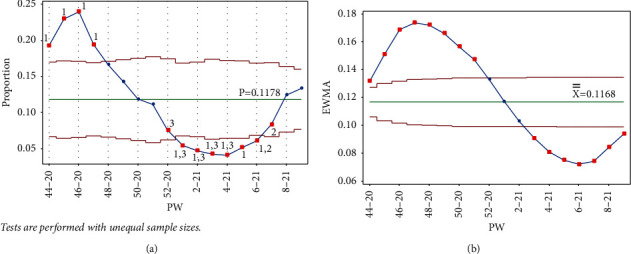
PW 9–21. (a) Laney p' control chart and (b) EWMA chart.

Test 2 was not triggered with the addition of PW 8–21, and the Laney p' control chart monitored big jumps above the mean (Figure 10(a)). The EWMA chart still triggers a test 1 signal from the LCL side. The government officially announced the second wave of the pandemic in the same week. Restriction measures were reintroduced, most notably a nationwide Friday curfew from Thursday 10 : 00 p.m. until Saturday 6 : 00 a.m., excluding the time of Friday prayers, and a weekday nighttime curfew was extended to between 10 : 00 p.m. and 6 : 00 a.m.

**Figure 11:**
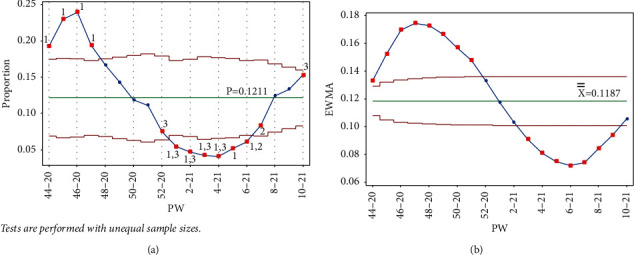
PW 10–21. (a) Laney p' control chart and (b) EWMA chart.

We can see that timely countermeasures and actions could have helped prevent escalation if control charts had been employed earlier, especially as signals started showing in PW 5–21, at least three weeks before the government took any action. With PW 9–21, the IR continued rising. Adding PW 10–21 to the Laney p' control chart triggered the upward test signal (test 3) when the government reverted to online teaching for all grades (Figure 11(a)). Test 1 was not triggered with the addition of PW 10–21 to the EWMA chart (Figure 11(b)).

**Figure 12:**
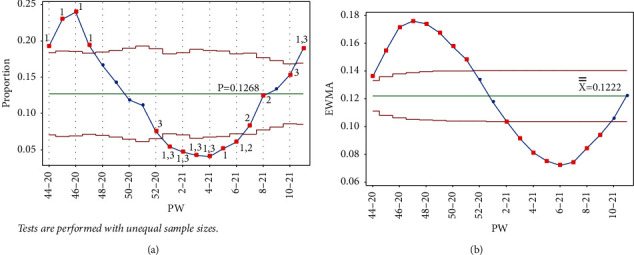
PW 11–21. (a) Laney p' control chart and (b) EWMA chart.

The addition of PW 11–21 to the Laney p' control chart triggered tests 1 and 3 after the government extended the curfew by 3 hours, to start at 7 : 00 pm. The upward period leads to the second peak in PW 11–21, as shown in Figure 12(a).

**Figure 13:**
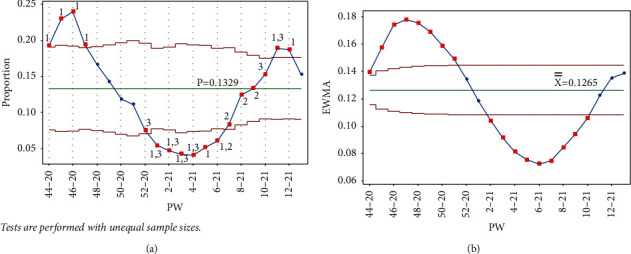
PW 13–21. (a) Laney p' control chart and (b) EWMA chart.

After adding PW 12–21 to the Laney p' control chart, test 3 was not triggered, but test 1 was, indicating that the IR had slowly started to change direction. PW 13–21 did not trigger any test, indicating a faster decrease in the IR, as shown in Figure 13(a). The EWMA is still showing an increase in the IR (Figure 13(b)).

**Figure 14:**
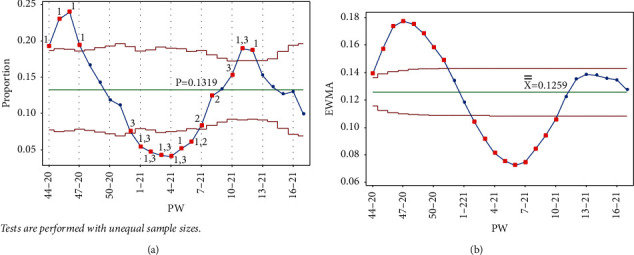
PW 17–21. (a) Laney p' control chart and (b) EWMA chart.

The period from PW 12–21 to PW 20–21 showed a decline in the IR, resulting from the government maintaining the curfew until PW 17–21. Furthermore, the customary large gatherings of people for the congregational evening prayers (Taraweeh), usually held in Ramadan, the fasting month for Muslims during this period (PW 15–21 to PW 19–21), were not permitted. Then in PW 17–21, the government relaxed some of the restrictions, lifting the Friday curfew, so the Taraweeh prayers were allowed and public parks reopened. In addition, the government reduced the number of curfew hours during the 3-day Eid al-Fitr feast that marks the end of Ramadan, when families and friends visit each other (Figure 14).

**Figure 15:**
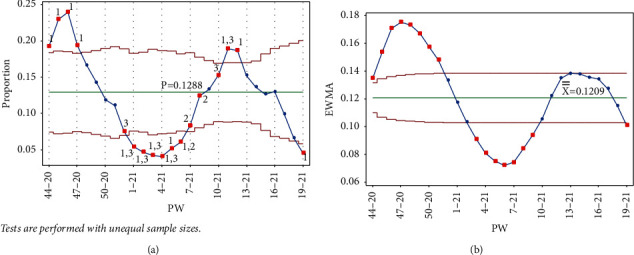
PW 19–21. (a) Laney p' control chart and (b) EWMA chart.

A limited number of cases of the Brazilian variant were found in Jordan in PW 12–21, but, as Figure 15 shows, the variant did not spread in the country.

Similarly, although the cases of the Delta variant were first reported in PW 18–21, a relatively stable period and a low number of new cases between PW 21–21 and PW 37–21 were observed.

Test 1 was triggered from the LCL for all points PW 19–21 to 24–21.

In PW 25–21, besides test 1, test 2 was also triggered. In this week, fully vaccinated people were exempted from the weekday night curfew. In PW 28–21, the government also reduced the night curfew for the nonvaccinated.

**Figure 16:**
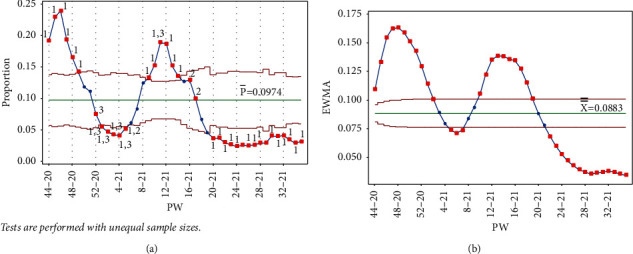
PW 35–21. (a) Laney p' control chart and (b) EWMA chart.

Another religious feast came during PW 29–21 (Eid al-Adha), when families and friends visit each other. This caused a slight rise in the IR in the period from PW 30–21 to PW 32–21. No test was triggered by this slight rise (Figure 16). In PW 35–21, the government announced the end of the curfew. Commercial stores and leisure facilities reopened in the same week.

**Figure 17:**
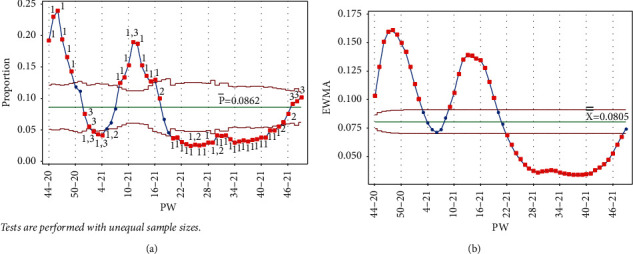
Upward period, third peak (PW 49–21). (a) Laney p' control chart and (b) EWMA chart.

From September 22 to October 2, 2021 (from PW 38–21 to PW 40–21), large numbers of people attended the Jerash culture festival. A huge debate ensued in the local and social media on the negative effect these gatherings were expected to have on the IR. To make matters worse, four concerts were held between PW 40–21 and PW 44–21 in different cities in Jordan. For the Laney p' control chart, tests 1 and 2 were triggered for all the points from PW 24–21 to PW 42–21. When PW 43–21 was added, in addition to tests 1 and 2, test 3 was also triggered, indicating a rise in the IR. Adding points PW 44–21 and PW 45–21 again triggered tests 1, 2, and 3. Between PW 46–21 and PW 49–21, tests 2 and 3 were triggered, showing an upward movement to the third peak in PW 49–21, as shown in Figure 17(a). The EWMA chart only after adding PW 49–21 did not trigger test 1 (Figure 17(b)).

One reason for the rise in the IR may have been the easing of some of the restrictions, especially the effect of the Jerash festival and the concerts.

**Figure 18:**
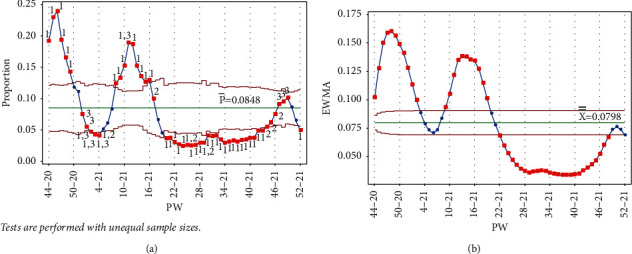
PW 52–21. (a) Laney p' control chart and (b) EWMA chart.

A downward trend was subsequently observed, from PW 50–21 to PW 52–21, where test 1 was triggered from the LCL side, as shown in Figure 18(a), indicating a sharp drop in the IR.

**Figure 19:**
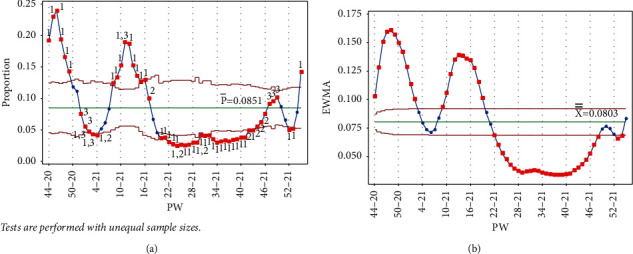
PW 9–22. (a) Laney p' control chart and (b) EWMA chart.

Adding PW 1–22 triggered test 1, and although adding PW 2–22 did not trigger any test, the point is in the in-control region of the chart, indicating that the IR was increasing, as seen in Figure 19(a). Adding PW 3–22 triggered test 1, but this time from the UCL side, indicating a large increase in the IR (Figure 19(a)). The EWMA chart did not trigger test 1. This rise followed the discovery of the first Omicron variant cases in Jordan, announced in PW 49–21.

**Figure 20:**
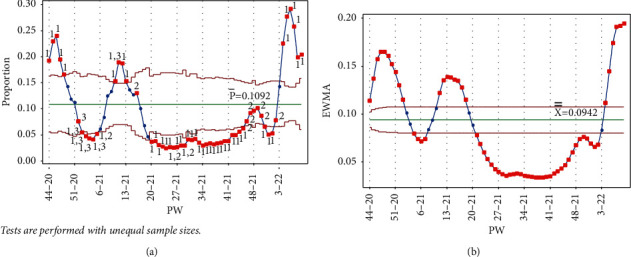
PW 9–22. (a) Laney p' control chart and (b) EWMA chart.

For the Laney p' control chart, adding PW 4–22 and PW 5–22 both triggered test 1. PW 5–22 also signaled test 2 in PW 46–21 and PW 47–21. The effect of adding this point on the mean is evident; the mean increased dramatically, and PW 46–21 and 47–21 points are below the mean as a result (Figure 20).

Likewise, adding PW 6–22, PW 7–22, and PW 8–22 also triggered test 1 and caused PW 48–21, 49–21, and 50–21 to signal test 2, respectively. Finally, adding PW 9–22 triggered test 1. In the EWMA chart, adding the points PW 4-22–PW 9–22 triggered test 1.

### 3.2. Test 3 Missed Signals

The discussion in this section is limited to the Laney p' control chart since test 3 applied does not apply to the EWMA chart.

There were three occasions in the analysis when a trend was observed, but test 3 was not triggered.

**Figure 21:**
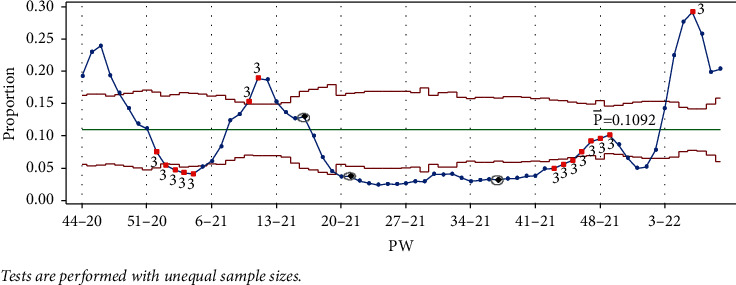
Test 3 missed signals.

A downward period starting from PW 12–21 would have triggered test 3 in PW 17–21, except that PW 16–21 (IR = 12.96%) was slightly higher than PW 15–21 (IR = 12.63%), as seen in Figure 21. The second occasion was in PW 21–21 (IR = 3.7%), which was slightly higher than in PW 20–21 (IR = 3.64%). Although a drop is visually clear in both cases, it was missed by test 3 (see Figure21).

The third occasion was the upward period from PW 34–21 to PW 49–21. Although test 3 signaled on PW 43–21, it could also have triggered on PW 40–21, but was delayed because PW 37–21 (IR 3.12%) was lower than PW 36–21 (IR 3.26%). The opportunity to take some earlier restrictive actions was lost, and the rise in the IR continued until PW 49–21. On all three occasions, test 3 failed, but a trend was clearly identified.

### 3.3. Multistage Analysis

We treated all the data points as a single stage when monitoring the IR, with the centerline and the upper and lower control limits recalculated with each successive data point added to the control chart. As is typical in process improvement projects, the plotted data involved two parts: that is, data corresponding to the current state, and data corresponding to the improved future state, after interventions and corrective actions were implemented.

We then attempted to use the concept of stages in a fresh approach to see whether any new perspectives could be gained from the analysis. Triggering test rule 1 depends on the value of the point with regard to the UCL and LCL. Similarly, triggering test 2 depends on the value of the point with regard to the CL. Triggering test 3 may also be affected since counting the number of points may be interrupted by starting a new stage. The UCL, LCL, and CL are recalculated using only the new data points that belong to the stage.

A stage ends when the point added causes a test to be signaled for the same point.

Stage 1 contained points up to PW 52–20, where signals were triggered for tests 1 and 3, indicating a fall in the rate, as shown in Figure 7(a).

**Figure 22:**
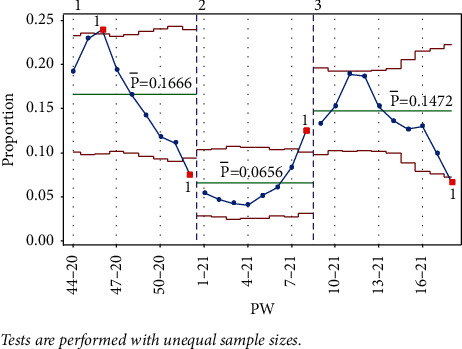
Stages 1–3 (the Laney p' control chart).

Starting stage 2 from PW 1–21 and adding the points to PW 8–21, test 1 was signaled on PW 8–21 from the UCL, indicating a rise. After adding more points to the chart starting at stage 3, the next signal was test 1 on PW 18–21, signaling a decrease, and ending stage 3, as shown in Figure 22.

**Figure 23:**
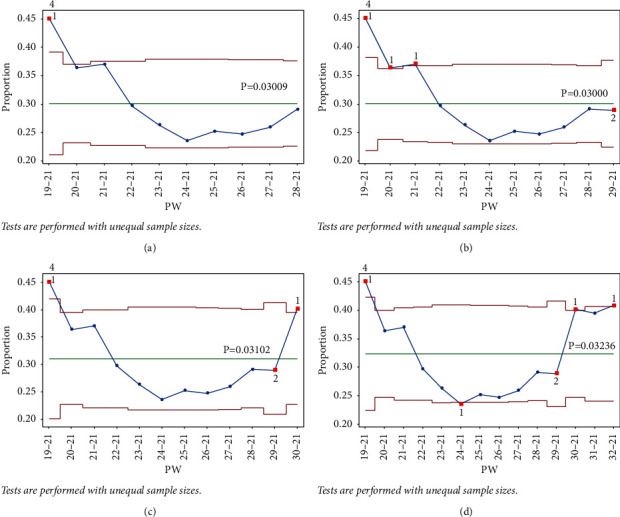
Stage 4.

After adding points to the chart up to PW 28–21 in stage 4, no signals were observed (Figure 23(a)). Adding point PW 29–21 signaled for test 2 (8 consecutive points under the centerline), however, and PW 20–21 and PW 21–21 signaled for test 1 from the UCL, as shown in Figure 23(b).

Next, adding PW 30–21 to the same stage signaled test 1 from the UCL, which was not signaled in the single-stage analysis. This indicated an increase in IR (Figure 23(c). Adding PW 31–21 did not signal any test, whereas adding 32–21 signaled test 1 (Figure 23(d)). However, since signaling a test marks the end of a stage, PW 30–21 starts stage 5.

In stage 5, points were added up to PW 41–21 with no signals. However, PW 42–21 signaled test 1 from the UCL, indicating an increase in the IR.

**Figure 24:**
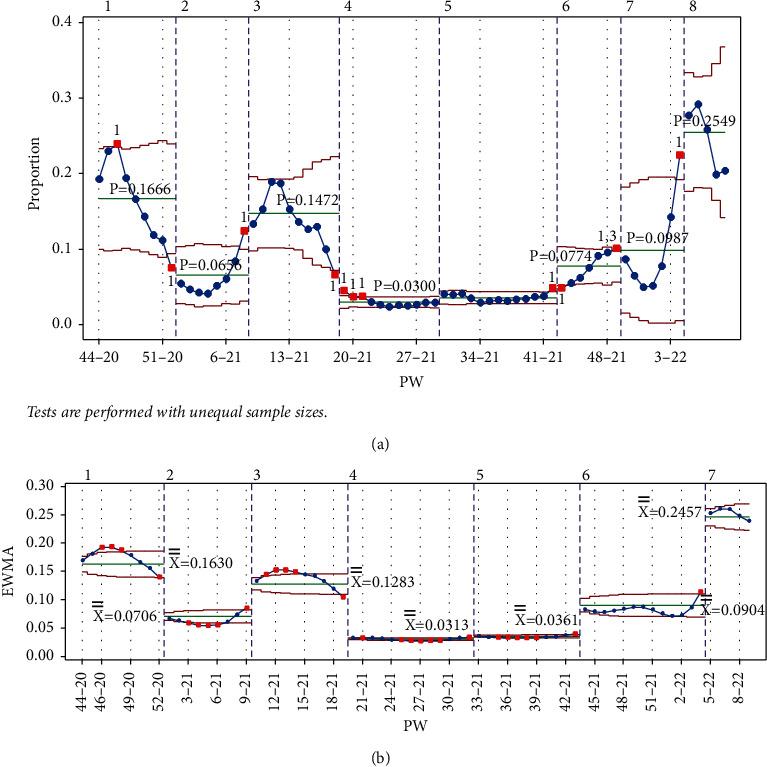
Stages 1–8. (a) Laney p' control chart and (b) EWMA chart.

Starting stage 6 from PW 43–21 and adding the points at PW 49–21 signaled tests 1 and 3, as shown in Figure 24(a). Starting stage 7 and adding points from PW 50–21 to PW 3–22 triggered no test signals. PW 4–22 signaled test 1 from the UCL, ending stage 7, and starting stage 8.

Comparing the analysis carried out in the multistage with that carried out in the single stage for the Laney p' control chart, one can see that the control chart is more sensitive to small changes in the IR when using the multistage analysis.

## 4. Conclusions

In this paper, we show the use of control charts in monitoring the progression of COVID-19 cases in Jordan.

If the control charts had been used, some early signs would not have been missed. In PW 5–21, for example, early signs of rising IR were detected, but no action was taken until PW 8–21. The charts would have allowed the government to take the appropriate actions more quickly and effectively.

The charts have proved useful in enabling us to study the effect of government actions designed either to restrict or to ease the social activities allowed. Furthermore, the charts confirmed that, as expected, restrictions effectively decreased the infection rate, whereas relaxing the restrictions proved to increase the rate.

This approach differs from the more traditional approach adopted in process improvement projects. The single-stage analysis gives the complete and overall picture of how the infection rate is changing, whereas the multistage analysis is more sensitive and reveals the smaller changes. Therefore, we recommend analyzing the data points both at the single stage and at the multistage.

Typically, test signals are considered indicators of a change in the process. However, we have seen that in some situations, the lack of a signal can also indicate a change in the process. Further, adding points to the chart may trigger a signal on the same point in some cases, whereas in other cases, it may trigger a signal on other previous points.

Variants of tests 2 and 3 are not implemented in the SPC software; we therefore invite software developers to include these invaluable tests.

Comparing the performance of tests 1 and 3 in detecting process change, one can see that no test outperformed the other, as shown in Table 3. The downward period (1), for example, was identified by both tests in PW 52–20. Using the test rules in the typical way, the upward period (2) was identified by test 3 one week earlier than by test 1. However, when using the test rule in the nontypical way, i.e., when the test does not signal, it is clear that test 3 identified the process change in PW 5–21, five weeks earlier. The downward period (3) was identified by test 1 in PW 19–21, but if the variant of test 3 had been used, the downward trend would have been identified earlier in PW 17–21.

The upward period (4) was missed by all tests in the single-stage analysis but was detected by test 1 in PW 30–21 using the multistage analysis. The downward period (6) was identified only by test 1 in PW 52–21. The upward period (7) was first identified by test 1 in PW 3–22.

When comparing the Laney p' control chart with the EWMA control chart, the EWMA chart signaled the downward period (1) in Table 3, one week earlier than the Laney p' control chart. In other downward or upward changes of direction (2, 3, 5, and 6), Laney p' control chart outperformed the EWMA chart. Both control charts signaled in the same week for a change of direction (7).

## Figures and Tables

**Table 1:** Sensitizing rules [63].

Rule
One or more points outside of the control limits
Two of three consecutive points outside the two-sigma warning limits but still inside the control limits
Six points in a row steadily increasing or decreasing
Four of five consecutive points beyond the one-sigma limits
A run of eight consecutive points on one side of the centerline
Fifteen points in a row in zone C (both above and below the centerline)
Fourteen points in a row, alternating up and down
Eight points in a row on both sides of the centerline with none in zone C
An unusual or nonrandom pattern in the data
One or more points near a warning or control limit

**Table 2:** Test rules for special causes [57].

Rule	Reference to Table 1
One or more points outside of the control limits	1
A run of eight consecutive points on one side of the centerline	5
Six points in a row steadily increasing or decreasing	3
Fourteen points in a row alternating up and down	7

**Table 3:** Comparison between tests 1 and 3 in detecting process change.

	Direction	Single stage				Multistage
		Laney p'			EWMA	Multistage
		Test 1	Test 3	Test 3^*∗*^		
1	Downward	PW 52–20	PW 52–20		PW 51–20	
2	Upward	PW 11–21	PW 10–21		PW 10–21^*∗∗*^	
		PW 7–21^*∗∗*^	PW 5–21^*∗∗*^			
3	Downward	PW 19–21		PW 17–21	PW 19–21	
4	Upward					PW 30–21 test 1
5	Upward	PW 46–21^*∗∗*^	PW 43–21	PW 37–21	PW 49–21^*∗∗*^	PW 40–21
6	Downward	PW 52–21				
7	Upward	PW 3–22	PW 6–22		PW 3–22^*∗∗*^	PW 4–22

^*∗*^test 3 variant, ^*∗∗*^ test did not trigger.

## Data Availability

All data used to support the findings of the study can be obtained from the author upon request.
